# Clinical risk stratification of stroke caregiver distress: an interpretable nonlinear model of caregiving-load categories

**DOI:** 10.3389/fpsyg.2026.1842020

**Published:** 2026-08-27

**Authors:** Yingjie Zheng, Shailing Ma, Xiaohui Liu, Yuyan Yang, Ru Gan, Jiajia Lai, Yijia Qi, Jing Li, Lijun Wang, Miaomiao Chen

**Affiliations:** 1 School of Nursing, Ningxia Medical University, Yinchuan, China; 2 Department of Emergency Medicine, General Hospital of Ningxia Medical University, Yinchuan, China; 3 Ningxia Health Vocational and Technical College, Shizuishan, China

**Keywords:** clinical risk stratification, coping, machine learning, psychological distress, SHAP, social support, stroke caregivers, XGBoost

## Abstract

**Background:**

Family caregivers of stroke survivors frequently experience psychological distress. Social support and active coping may be protective, but their associations can vary across levels of caregiving load. Interpretable machine learning may help characterize such nonlinear patterns without assuming a constant linear association.

**Methods:**

This multicenter cross-sectional study included 506 family caregivers. High psychological distress was defined as a Hospital Anxiety and Depression Scale (HADS) total score ≥15. We trained a machine-learning model using extreme gradient boosting (XGBoost) with six fixed analytic predictor domains: role burnout, perceived social support, active coping, daily sleep-duration category, daily caregiving-time category, and income level. Model performance was evaluated in a stratified 30% held-out test set, with bootstrap confidence intervals and nested cross-validation. TreeSHAP was used for model attribution and interaction exploration.

**Results:**

Among 506 caregivers, 178 (35.2%) met the high-distress criterion. Most caregivers were aged 18–59 years (85.4%), and 60.3% were women. XGBoost achieved a test-set AUC of 0.890 (95% CI: 0.830–0.937); the mean nested five-fold cross-validated AUC was 0.891 (SD 0.024). Random forest and support vector machine models showed comparable discrimination. Role burnout had the largest mean absolute SHAP value, followed by active coping and perceived social support. The three recorded sleep-duration categories ( ≤ 5, 6–8, and ≥9 h) and caregiving-time categories (6–8, 9–16, and ≥17 h) showed category-level differences in model attribution; these boundaries were questionnaire-defined and were not estimated as continuous thresholds. Interaction plots suggested that the associations of support and coping varied across load categories.

**Conclusions:**

An interpretable nonlinear model identified combinations of role burnout, psychosocial resources, and caregiving-load categories associated with high psychological distress. The category-level and interaction findings are hypothesis-generating rather than causal or diagnostic and require prospective external validation before clinical threshold use.

## 1 Introduction

Stroke-related disability can create sustained physical, emotional, and social demands for family caregivers. Caregiver burden and depressive symptoms are common across the recovery trajectory and can affect both members of the stroke-caregiver dyad (Grant et al., 2000; Cameron et al., 2014; Tziaka et al., 2024). Sleep disruption, extended caregiving time, limited resources, and the caregiver's own appraisal of the role may contribute to distress, although their relationships are likely to differ across individuals and contexts (Kulkarni et al., 2025; Oh et al., 2024).

Perceived social support and active coping are consistently associated with better caregiver adjustment (Grant et al., 2006; Huang et al., 2009; Liu et al., 2025). Interventions therefore commonly incorporate education, problem solving, psychological support, and dyadic or remote-care components (Bakas et al., 2022; Panzeri et al., 2019; Sun et al., 2023). However, the magnitude of these associations may depend on concurrent caregiving demands. A modeling strategy that allows nonlinear and interactive patterns may complement conventional descriptive analyses.

Machine-learning approaches have increasingly been used for caregiver and mental-health risk stratification (Conte et al., 2024; Bozorgmehr and Weltermann, 2023; Du et al., 2025; Shi et al., 2025). XGBoost can represent nonlinearities and interactions in tabular data (Chen and Guestrin, 2016), while SHapley Additive exPlanations (SHAP) can summarize how variables contribute to a fitted model's output (Lundberg and Lee, 2017). These tools are useful for prediction and exploratory interpretation, but SHAP values do not establish causality, and apparent category changes should not be treated as biologically estimated cut points when the underlying variables were collected categorically.

Accordingly, this study aimed to (1) develop and internally evaluate an interpretable model for HADS-defined high psychological distress among stroke caregivers; (2) describe the relative model contributions of role burnout, social support, coping, sleep category, caregiving-time category, and income; and (3) explore whether support- and coping-related model attributions differed across the recorded load categories. We treated all category-level and interaction results as associative and hypothesis-generating.

## 2 Methods

### 2.1 Study design and participants

This multicenter cross-sectional study used convenience sampling to recruit primary family caregivers of stroke survivors from rehabilitation departments and neurology units at five integrated healthcare institutions in Yinchuan and Wuzhong, Ningxia, China, between July 2022 and January 2023.

Sample adequacy was assessed pragmatically before modeling. The final model used six fixed analytic predictor domains and included 178 high-distress events, corresponding to approximately 29.7 events per predictor domain. Under a conventional planning value of 10 events per domain and an anticipated event proportion of 35%, at least 172 participants would have been required (6 × 10/0.35); the achieved sample of 506 exceeded this planning value. We recognize that this heuristic is not a substitute for external validation, particularly for a nonlinear model (Riley et al., 2020).

Caregivers were eligible if they (1) were the primary unpaid family member responsible for the patient's daily care; (2) cared for a survivor with ischemic or hemorrhagic stroke confirmed by CT or MRI; (3) had provided care for at least one month; and (4) could understand and complete the survey. Caregivers were excluded if they were paid care staff, had a documented severe psychiatric disorder or cognitive impairment predating the stroke, or had more than 10% missing data in a core scale.

**Figure 1:**
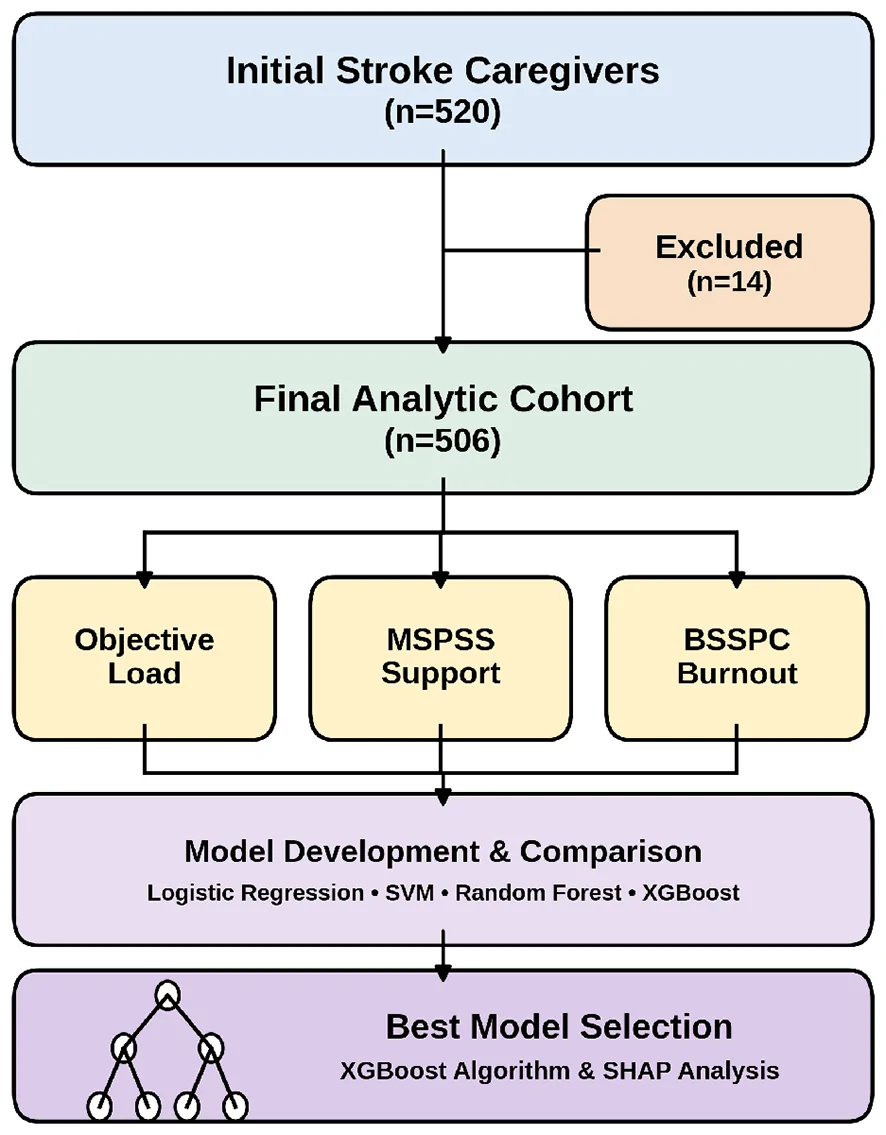
Integrated study flowchart showing participant recruitment, formation of the final analytic cohort, a simplified overview of the measured input domains, comparison of logistic regression, support vector machine, random forest, and XGBoost, and subsequent XGBoost/TreeSHAP interpretation. The three middle boxes are schematic and do not enumerate all six predictors; active coping and income were also included in the full model as specified in the Methods. In the figure, “Best Model Selection” denotes selection of XGBoost as the primary model for interpretation because of its compatibility with native TreeSHAP; it does not indicate statistically superior discrimination over random forest or SVM.

A total of 520 questionnaires were distributed. Fourteen records were excluded because of incomplete core assessments, withdrawal, or failure to meet eligibility, missingness, range, or logical-consistency criteria, leaving 506 observations (effective response rate, 97.31%). The participant-selection and analysis workflow is summarized in Figure 1.

### 2.2 Data collection procedures

Trained clinical researchers at each participating site approached eligible caregiver–patient dyads during the survivor's hospitalization. After written informed consent, caregivers completed the study questionnaires in person; questionnaire responses, including sleep and caregiving-time categories, were self-reported. Caregiver questionnaire data were collected directly from participants and were not retrospectively extracted from the electronic medical record. Stroke diagnosis and relevant patient information were checked against the medical record. Quality control occurred at two stages: site researchers reviewed questionnaire completeness before the encounter ended, and eligibility, missingness, range, and logical-consistency checks were applied before the frozen analysis file was exported. Records were de-identified using study codes and entered into the study database before dataset-level checks and export. Participant-facing data collection was completed before the machine-learning workflow began.

The protocol was approved by the Ethics Committee of Ningxia Medical University (No. 2022-017) and conducted in accordance with the Declaration of Helsinki. All participants provided written informed consent.

### 2.3 Measures

#### 2.3.1 Caregiver characteristics and load categories

The questionnaire recorded caregiver age, gender, education, employment, marital status, relationship to the patient, and household income, together with patient characteristics. Daily sleep duration was recorded only as ≤ 5, 6–8, or ≥9 h, and daily caregiving time only as 6–8, 9–16, or ≥17 h. These are prespecified questionnaire categories; exact hours within a category were not available, and the category boundaries were therefore not estimated by the model.

#### 2.3.2 High psychological distress

Psychological distress was assessed with the 14-item Hospital Anxiety and Depression Scale (HADS), comprising seven anxiety and seven depression items scored from 0 to 3 (Zigmond and Snaith, 1983). The total score ranges from 0 to 42. Consistent with prior use of the total HADS as a distress-screening measure, a total score ≥15 defined the high-distress group (Wang et al., 2011). This label is a screening-based analytic outcome and not a psychiatric diagnosis. Cronbach's α for the total HADS in this sample was 0.88.

#### 2.3.3 Caregiver burnout

Caregiver burnout was measured using the 24-item Burnout Scale for Stroke Patients' Caregivers (BSSPC), developed for Chinese stroke caregivers and comprising role strain, physiological exhaustion, psychological stress, and social isolation dimensions (Gan et al., 2025). Items are scored on a five-point Likert scale, with higher scores indicating greater caregiver burnout. The role-burnout dimension was included in the prediction model; the total score was used for descriptive group comparisons. Cronbach's α for the total BSSPC was 0.91.

#### 2.3.4 Perceived social support and coping

The 12-item Multidimensional Scale of Perceived Social Support (MSPSS) assessed perceived support from family, friends, and significant others (Zimet et al., 1988). The Trait Coping Style Questionnaire (TCSQ) assessed active and negative coping tendencies; the active-coping subscale was included for modeling (Jiang and Zhu, 1999). In this study, Cronbach's α values were 0.82 and 0.80 for the active- and negative-coping subscales, respectively.

### 2.4 Statistical analysis and model development

Group comparisons used Pearson's chi-square test for categorical variables and Welch's independent-samples *t* test for continuous scores. All tests were two-sided, and *p* < 0.05 was considered statistically significant.

**Figure 2:**
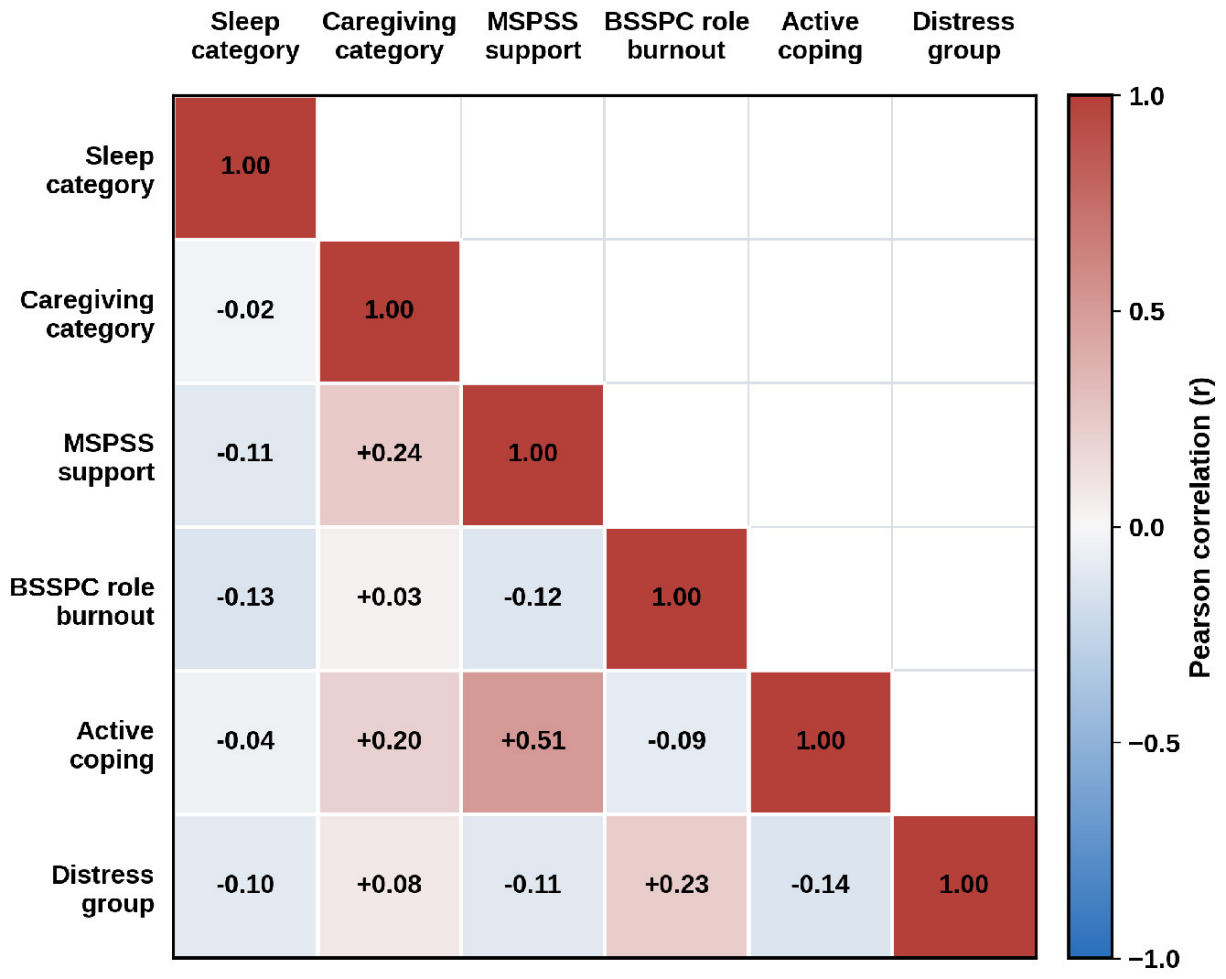
Lower-triangle Pearson correlation matrix for the fixed analysis variables and binary outcome. The observed correlation between role burnout and the binary high-distress outcome was modest rather than near unity.

The frozen, de-identified comma-separated analysis file was imported into an author-controlled Python pipeline. The primary model contained exactly six predictors: BSSPC role burnout, MSPSS total support, TCSQ active coping, daily sleep-duration category, daily caregiving-time category, and income level. For the reported analysis, this six-predictor set was fixed before model fitting and was not altered according to held-out test-set performance. The final analytic fields contained no missing values. Continuous predictors were standardized and categorical predictors were one-hot encoded within each training fold, preventing information from the validation or test observations from entering preprocessing. For example, MSPSS, TCSQ active-coping, and BSSPC role-burnout scores were standardized using training-fold statistics, whereas the recorded sleep-duration, caregiving-time, and income categories were represented by indicator variables fitted within each training fold. Figure 2 reports the Pearson correlations of the analysis variables as a transparency check; no predictor pair had |*r*|>0.75.

The data were divided once into a stratified 70% training set (*n* = 354; 125 high-distress events) and 30% held-out test set (*n* = 152; 53 events) using random seed 20260329. XGBoost hyperparameters were selected within the training set by five-fold stratified grid search optimizing AUC. The grid included 100, 150, or 200 trees; learning rates of 0.05 or 0.07; maximum depths of 2 or 3; minimum child weight of 1; subsample of 0.8; and column subsample of 0.8. The selected XGBoost configuration used 150 trees, learning rate 0.07, maximum depth 3, and the remaining fixed values above.

Random forest, radial-basis-function support vector machine (SVM), and logistic regression were fitted as compact reference benchmarks using the same split and fold-contained preprocessing. XGBoost was retained as the primary interpretation model because native TreeSHAP provides a consistent decomposition of tree-ensemble output. Accordingly, the “Best Model Selection” wording in the schematic workflow refers to interpretability-based selection for the planned SHAP analysis and not to a statistically superior AUC; the comparative results are reported without a superiority claim.

Test-set AUC was reported with a 95% percentile interval from 1,000 bootstrap resamples. Accuracy, sensitivity, specificity, and F1 score used a probability threshold chosen solely from out-of-fold predictions in the training set by maximizing Youden's index. As an additional internal-validation check, XGBoost underwent nested five-fold cross-validation, with four-fold tuning inside each outer fold. All analyses used Python 3.12.13, scikit-learn 1.8.0, and XGBoost 3.3.0; the complete scripts, fixed seed, parameter grids, figure-source data, and validation reports accompany the revision.

### 2.5 Model interpretation

TreeSHAP contributions and native TreeSHAP interactions were calculated for held-out test observations (Lundberg and Lee, 2017). Mean absolute SHAP values summarized global attribution. Sleep and caregiving-time results were plotted at the three levels actually recorded. Individual observations, category means, and 95% confidence intervals were shown; no interpolation to unobserved hours and no continuous threshold estimation were performed. Interaction figures display individual interaction values and equal-frequency-bin summaries within each category. Connecting lines are visual guides between observed summaries and are not fitted threshold curves. Because the design was cross-sectional and SHAP explains a predictive model rather than a causal mechanism, interpretations were restricted to associations with model output.

## 3 Results

### 3.1 Participant characteristics

Of 506 caregivers, 178 (35.18%) had a HADS total score ≥15. Age, gender, and education distributions did not differ significantly between the high- and lower-distress groups (Table 1). The corrected education comparison was χ^2^ = 2.621, *p* = 0.454.

**Table 1:** Baseline characteristics by HADS-defined psychological-distress group (*N* = 506).

Variables	Total	High distress (*n* = 178)	Lower distress (*n* = 328)	χ^2^	*p*-value
**Caregiver age**, ***n*** **(%)**				2.568	0.277
18–44 years	205 (40.5)	76 (42.7)	129 (39.3)		
45–59 years	227 (44.9)	82 (46.1)	145 (44.2)		
≥60 years	74 (14.6)	20 (11.2)	54 (16.5)		
**Caregiver gender**, ***n*** **(%)**				0.000	1.000
Male	201 (39.7)	71 (39.9)	130 (39.6)		
Female	305 (60.3)	107 (60.1)	198 (60.4)		
**Education**, ***n*** **(%)**				2.621	0.454
Primary school or below	112 (22.1)	46 (25.8)	66 (20.1)		
Junior high school	168 (33.2)	58 (32.6)	110 (33.5)		
High school/vocational	122 (24.1)	38 (21.3)	84 (25.6)		
College or above	104 (20.6)	36 (20.2)	68 (20.7)		

BSSPC burnout, MSPSS support, active coping, and negative coping scores differed between groups (Table 2). The overall distributions of caregiving-time categories (*p* = 0.148) and sleep-duration categories (*p* = 0.080) did not reach the prespecified univariate significance level. The subsequent machine-learning results therefore describe multivariable predictive attribution and should not be read as confirmation of independent category effects.

**Table 2:** Caregiving-load categories and psychometric scores by distress group.

Variables	Total	High distress (*n* = 178)	Lower distress (*n* = 328)	χ^2^/*t*	*p*-value
**Daily caregiving time**, ***n*** **(%)**				3.815	0.148
6–8 h	167 (33.0)	53 (29.8)	114 (34.8)		
9–16 h	255 (50.4)	88 (49.4)	167 (50.9)		
≥17 h	84 (16.6)	37 (20.8)	47 (14.3)		
**Daily sleep duration**, ***n*** **(%)**				5.042	0.080
≤ 5 h	149 (29.4)	61 (34.3)	88 (26.8)		
6–8 h	267 (52.8)	93 (52.2)	174 (53.0)		
≥9 h	90 (17.8)	24 (13.5)	66 (20.1)		
BSSPC burnout score	72.83 ± 14.32	76.75 ± 15.94	70.70 ± 12.89	4.350	< 0.001
MSPSS support score	47.80 ± 7.74	46.61 ± 8.41	48.44 ± 7.28	−2.448	0.015
Active coping (TCSQ)	29.65 ± 4.47	28.81 ± 5.13	30.10 ± 4.00	−2.910	0.004
Negative coping (TCSQ)	29.84 ± 4.77	30.57 ± 5.21	29.45 ± 4.47	2.425	0.016

### 3.2 Predictive performance

**Figure 3:**
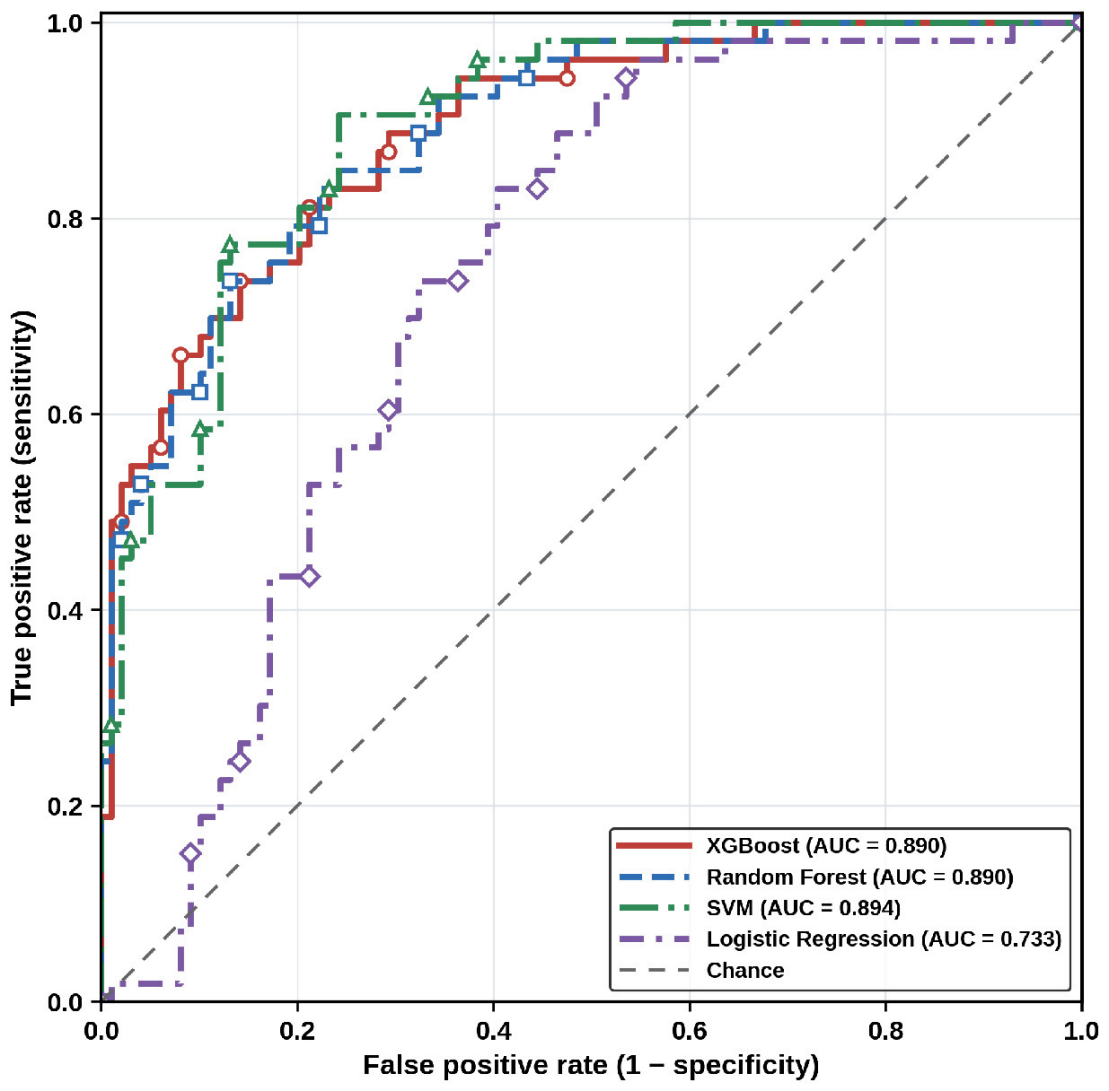
Empirical held-out test-set receiver operating characteristic curves. Open nodes denote observed ROC coordinates; curves were not smoothed.

On the held-out test set, XGBoost achieved an AUC of 0.890 (95% CI: 0.830–0.937), accuracy of 0.789, sensitivity of 0.755, specificity of 0.808, and F1 score of 0.714 (Table 3; Figure 3). The nested five-fold AUC was 0.891 (SD 0.024; fold range 0.852–0.917). Random forest (AUC 0.890) and SVM (AUC 0.894) had comparable discrimination; paired bootstrap intervals for XGBoost minus random forest (−0.022 to 0.021) and XGBoost minus SVM (−0.036 to 0.029) included zero. We therefore make no claim that XGBoost was superior to these nonlinear benchmarks.

**Table 3:** Held-out test-set model performance.

Model	AUC (95% bootstrap CI)	Accuracy	Sensitivity	Specificity	F1 score
XGBoost	0.890 (0.830–0.937)	0.789	0.755	0.808	0.714
Random forest	0.890 (0.829–0.938)	0.809	0.736	0.848	0.729
SVM	0.894 (0.840–0.938)	0.796	0.774	0.808	0.726
Logistic regression	0.733 (0.656–0.814)	0.658	0.849	0.556	0.634

### 3.3 Global model attribution

**Figure 4:**
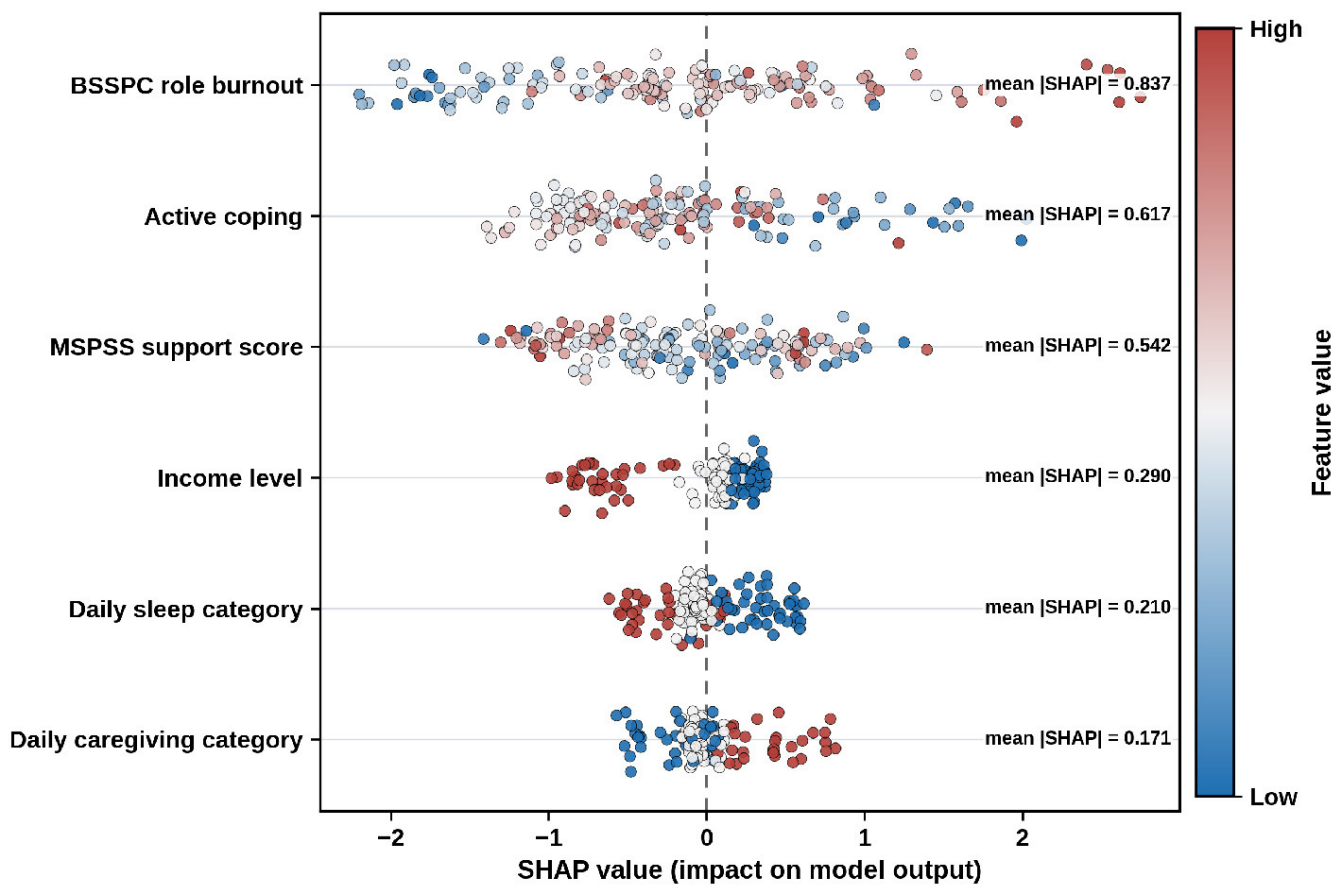
TreeSHAP summary plot for held-out test observations. Each point represents one caregiver; color denotes the observed feature value. SHAP values describe contribution to model output and not causal effects.

Role burnout had the largest mean absolute SHAP value (0.837), followed by active coping (0.617), perceived social support (0.542), income (0.290), sleep category (0.210), and caregiving-time category (0.171) (Figure 4). Higher role-burnout values generally contributed to higher model output, whereas the direction and magnitude of other contributions varied across observations.

Because role burnout is conceptually related to psychological distress, we conducted a sensitivity analysis that removed BSSPC role burnout and refitted XGBoost using the same split and tuning procedure. The reduced model achieved a test AUC of 0.735 (95% bootstrap CI: 0.644–0.811). Thus, role burnout contributed substantial predictive information, while the remaining five variables retained modest discrimination. The role-burnout/outcome correlation in Figure 2 was approximately *r* = 0.23, not 0.90.

### 3.4 Category-level load patterns

**Figure 5:**
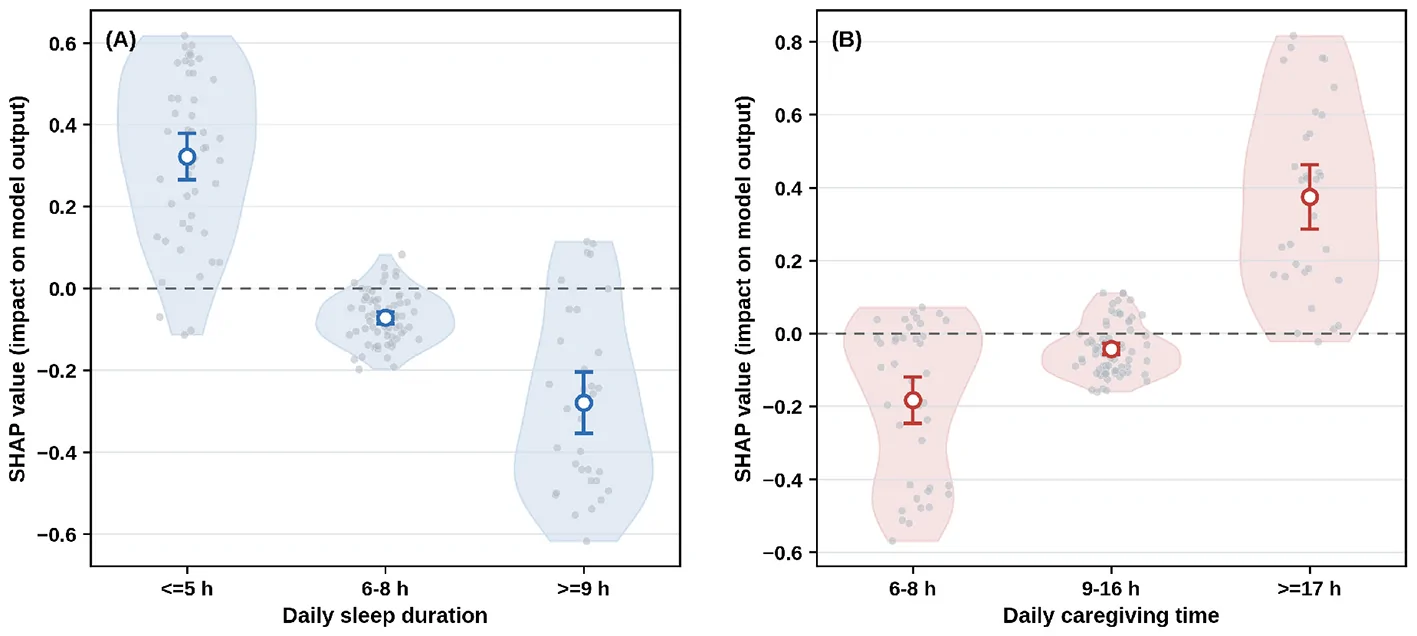
Category-level TreeSHAP distributions for **(A)** daily sleep duration and **(B)** daily caregiving time. Gray points are held-out observations; open nodes and whiskers are means and 95% confidence intervals. Categories are displayed exactly as recorded; no continuous cutoff is estimated.

Figure 5 replaces the former continuous-hour plots. The ≤ 5 h sleep category tended to have a higher mean sleep-related SHAP contribution than the longer-sleep categories, and the ≥ 17 h caregiving category tended to have a higher mean care-related contribution than the shorter-care categories. These are comparisons among the three recorded categories. They do not establish that risk changes precisely at 5 or 17 h, nor do they identify physiological tipping points.

### 3.5 Exploratory interactions

**Figure 6:**
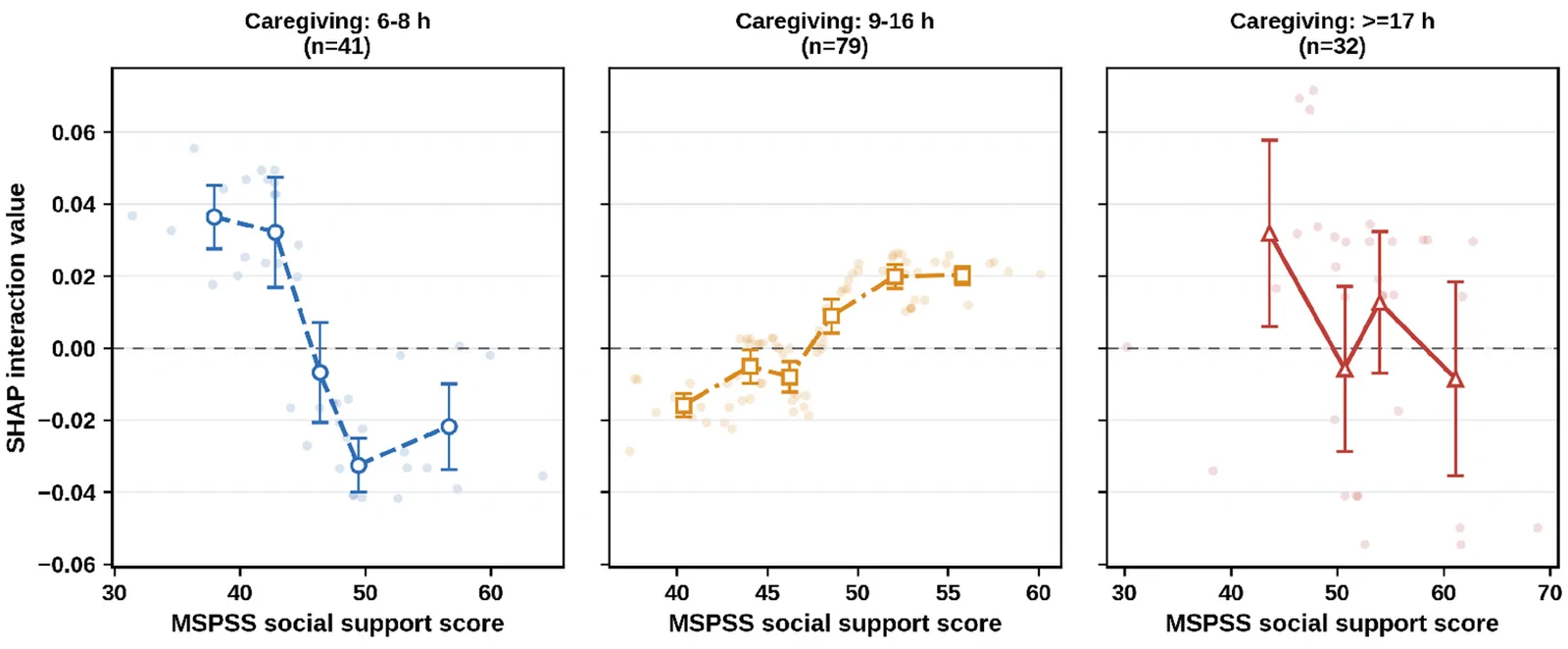
Native TreeSHAP interaction between perceived social support and the three recorded caregiving-time categories. Faint points are individual observations; open nodes, connecting lines, and whiskers show equal-frequency-bin means and 95% confidence intervals. Lines connect observed summaries only and are not continuous threshold estimates.

**Figure 7:**
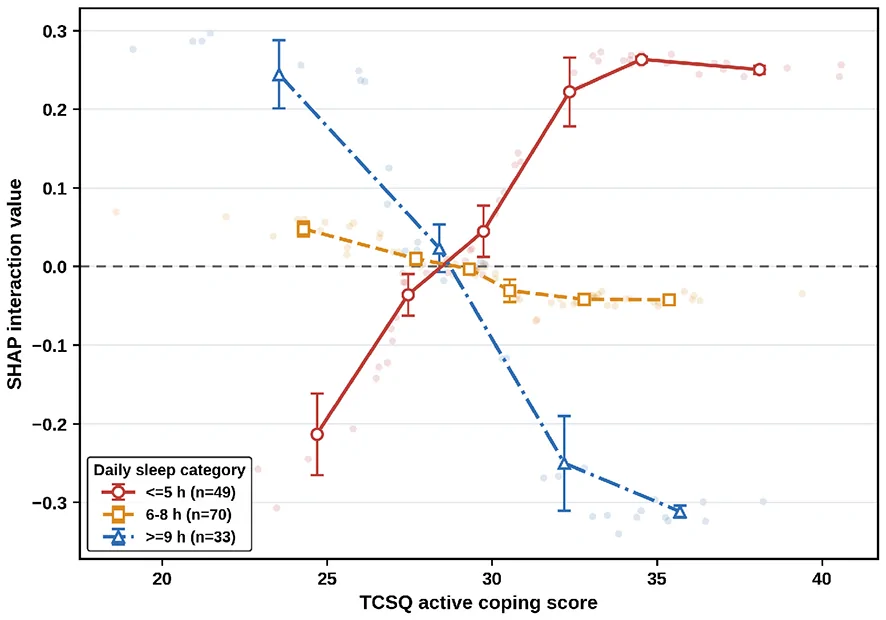
Native TreeSHAP interaction between active coping and the three recorded sleep-duration categories. Faint points are individual observations; open nodes, connecting lines, and whiskers show equal-frequency-bin means and 95% confidence intervals. Lines connect observed summaries only and are not continuous threshold estimates.

The support-by-caregiving-category and active-coping-by-sleep-category TreeSHAP interactions are shown in Figures 6, 7. The observed interaction contributions varied across category-specific ranges. These patterns suggest possible heterogeneity in model attribution, but they do not demonstrate that support or coping “fails,” becomes “neutralized,” or causes a change in distress. Sparse observations within some bins and the internal, cross-sectional design require cautious interpretation and independent longitudinal validation.

## 4 Discussion

This study developed an internally evaluated nonlinear prediction model for HADS-defined high psychological distress among stroke caregivers. The revised analysis identifies role burnout, active coping, perceived social support, income, and the recorded load categories as contributors to model output. XGBoost, random forest, and SVM showed similar test discrimination; XGBoost was used for interpretation because TreeSHAP is directly available for the fitted tree ensemble.

The category-level plots support a more limited interpretation than the original continuous curves. Caregivers in the shortest-sleep and longest-care categories tended to receive higher load-related model contributions, but the source questionnaire did not contain exact continuous hours. The labels ≤ 5 h and ≥17 h are therefore useful for describing the sampled categories, not for declaring universal biological boundaries. Moreover, their univariate group comparisons were not statistically significant, underscoring that the SHAP plots concern the fitted multivariable model rather than independent effects.

Perceived support and active coping remain clinically relevant resources (Grant et al., 2006; Bakas et al., 2022). The exploratory interactions indicate that their model contributions may differ across load categories, which could motivate prospective work examining when practical respite, sleep support, psychological support, or combined interventions are most useful. The present data cannot show temporal precedence or intervention efficacy; statements that physical load overrides, collapses, or neutralizes resilience have therefore been removed. After prospective external validation, the model could potentially help identify caregivers who warrant a more detailed psychosocial assessment; it should not be used as an autonomous diagnostic tool or as a mandatory treatment rule.

### 4.1 Strengths and limitations

Strengths include multicenter in-person recruitment, use of established psychological measures, a fixed compact predictor set, fold-contained preprocessing, a held-out test set, bootstrap uncertainty, nested cross-validation, a sensitivity analysis, and auditable figure-source data. The revised category plots also preserve the measurement scale actually collected.

Several limitations are important. First, this was a cross-sectional study, so reverse direction and residual confounding are plausible; prediction and SHAP attribution must not be interpreted as causation. Second, all performance estimates are internal, and convenience sampling from five institutions in one Chinese region limits transportability. Third, sleep and caregiving time were self-reported in broad categories; the study cannot estimate continuous dose-response curves or exact cutoffs. Fourth, the BSSPC was developed in the same overall caregiver cohort and role burnout is conceptually related to HADS distress. Although its observed outcome correlation was modest and the reduced-model sensitivity analysis is reported, this design may still increase apparent performance and requires external validation using an independently developed measurement workflow. Fifth, the sample-size calculation was a pragmatic planning heuristic rather than a formal guarantee against overfitting. Finally, the interaction findings are exploratory, category dependent, and require preregistered longitudinal replication.

## 5 Conclusion

A compact XGBoost model showed good internal discrimination for HADS-defined high psychological distress among stroke caregivers and provided interpretable category-level and interaction patterns. The ≤ 5 h sleep and ≥17 h caregiving labels are observed questionnaire categories, not model-derived physiological tipping points. These findings can inform hypotheses and future external validation, but they should not yet be used as causal claims, diagnostic rules, or mandatory clinical thresholds.

## Data Availability

The raw data supporting the conclusions of this article will be made available by the authors, without undue reservation.
